# Etiology, Infection Carriers, and Treatment of Typhoid Fever

**Published:** 1908-12

**Authors:** B. W. Hall

**Affiliations:** Bowman, Ga.


### ETIOLOGY, INFECTION CARRIERS, AND TREATMENT OF TYPHOID FEVER.*

BY B. W. HALL, M. D., BOWMAN, GA.

In surgery, the art and science of the practice of medicine, typhoid fever included, we are learning to unlearn former ideas and theories. The crude in all science, art and philosophy is rapidly having the search light turned on in order to establish elementary facts.

According to historical descriptions of fever in the days of hipocrates it was not ristorical descriptions of fever in the days of seventeenth century it was regarded more as a symptom of a distinct condition rather than as a disease. In France and England, during the latter part of the eighteenth and early part of the nineteenth century, typhoid fever became more fully understood as a disease separate and distinct from typhus fever. Its causes and pathological anatomy became more fully understood.

In this brief sketch of typhoid fever I will eliminate as much as possible theoretical knowledge except that which bears directly upon the establishment of commonly accepted elementary facts.

Guessing now, little clouds the way in the diagnosis of typhoid fever. The experienced bacteriologist and pathologist can usually speedily settle all doubt in the diagnosis. The state, municipal and national government have already made considerable progress in the eradication of typhoid and other fever infections

---

*Rea· before Eighth Congressional Medical Association held at Athens, Ga., August 19, 1908.

and still greater progress is possible by instituting legal and hygenic rules of more force than they ever have done hitherto.

Formally, when physicians did not known just how this disease was communicated, it was considered inevitable and every one expected to have it sooner or later. Many persons actually believed it served a good purpose in cleansing the system in the event the patient recovered.

It is now an undisputed fact that typhoid fever is caused by a specific bacillus which enters the system through the mouth and the only cleansing which takes place is done, not by the disease, but when nature and medicine clear the system of toxins and infection.

For many years it was thought that a chemic poison produced by certain putrefactive substances caused typhoid fever. The old putrefactive theory has some well grounded truths. But the general accepted idea now held is that there is a direct infection by a specific bacillus or organism, which is taken into the system through the water or food.

It is an agent of specific origin and specific action. No case of typhoid originates spontaneously, but every case has an antecedent cause. The typhoid germs may be spread by the patient himself. Hence disinfection of the stools is absolutely essential in order to stop the spread of the disease.

Among local causes may be mentioned, extensive excavations of earth, low level of ground water, polluted drinking water, infected milk or polluted food.

Carriers.—(1) water, (2) house fly, (3) food or milk, (4) persons themselves, (5) air, (6) dust, (7) unsanitary clothing.

A 10 per cent. solution of formaldyhyde will destroy the house fly, when place in rooms or in the dining rooms. Milk laws in cities will prevent infection from that source.

In cities where infections are known to exist, bottle water is always procurable by persons who can afford to buy. But the vast majority can not afford to buy table water when the full supply is contaminated, and the average citizen trusts to luck rather than take the trouble of providing against disaster. He declines to boil his drinking water even during the presence of an epidemic or when the health officer calls his attention to the necessity of doing so.

Typhoid fever is not prominent in the list of fatal diseases. The percentages of fatalities is unusually small, but the disease entails long illness, requires careful nursing and is a heavy expense to every family it attacks.

Unlike many infections, typhoid fever may come from the country to the city; from the large to the smaller villages. Careless nursing of one case may pollute the water supply of an adjacent town and produce an epidemic of large proportions.

Even after physicians discovered the means by which typhoid fever is communicated it was generally believed that water, free from visible impurities, was safe and that visible foul water was dangerous.

Although the latter conclusion is generally correct the former is wholly erroneous. When a city's water supply comes directly from a muddy stream the probabilities are that it contains the infection as typhoid fever is existent at all times and streams are open to pollution from many sources, but perfectly clear water having every appearance of purity may be more dangerous than muddy water. The house filter clearifies, but does not actually cleanse.

Bacteria may be eliminated from water only by an elaborate system of municipal filtration or by thorough boiling.

An illustration of the truth of this fact was offered recently when typhoid fever became prevalent in a fashionable quarter of an American city where bottled water was used almost exclusively for drinking. Investigation showed that the water came from a "spring" upon the property of the dealer. Bacteriologists discovered that the water contained typhoid baccilli and further investigation showed that the "spring" was a leak from a sewer.

It will be seen that the water was filtered through several yards of earth, had been made perfectly clear and apparently pure, without eliminating the bacteria.

This is as much as an ordinary house filter can be expected to do.

American triumphs in the Panama canal zone which in 1905 were hot beds of disease, are now, after three years' of hospital supervision, transformed into practically new cities, by means of applying well known hygenic health laws and modern hospitals, installing pure water and a well regulated sewerage system.

In the London Lancet of June 27, 1908, pleasure seekers are warned against the dangers of the deadly holidays spent away from cities. Among other things, the Lancet goes on to state that almost every detail of a holiday is emphasized except the all-important question of hygiene. In these deadly holidays, families are frequently plunged into death traps at seaside of country village resorts. The natives of these seaside or country village resorts have become immune to these infected vicinities. Whereas the holiday, pleasure seeker, not accustomed to the impure water and unsanitary conditions, quickly become infected.

Instances are not wanting where soldiers just passing through these infected areas have taken typhoid fever, whereas the natives were not the least affected with the disease.

The boy who lives over the unsanitary stable is often healthy enough, but this method of living suddenly introduced to a person used to a carefully guarded sanitary environment, might prove to be a serious menace to his health.

In the City of Pittsburg, Penn., after thoroughly purifying the water supply and disinfecting all known causes of typhoid fever, an elderly lady who six years previously had been in an infected district communicated the disease to another family.

In the City of Georgetown, a suberb of Washington, D. C., a patient communicated the desease this summer after having acted as carrier 18 years. Further investigation on this point develops the fact that two per cent. of all convalescent cases of typhoid acts as carriers.

Individuals living in an infected location, may develop fever one summer, while others will escape and then may develop fever the summer following. An infected, vacated house frequently carries fever one year later to the incoming family.

As previously stated, a specific bacteria of direct transmission, demonstrated by pathologist and bacteriologist, has become the accepted etiological factor in the development of typhoid fever.

Theorizing from cause to effect Elberth in 1880 discovered the baccillus typhosus. Gaffky and many other bacteriologists have confirmed the discoveries of Eberth. Gaffky was the first to present a firm foundation for bacteriological isolation and growing of pure culture from healthy individuals to formulate the definite conclusion that the organism is the specific cause of typhoid fever.

The baccilli of Elberth in their ordinary form, are short, thick rods with rounded extremeties and about thrice as long as they are wide and in length are one-third the diameter of a red blood corpuscle.

From recent scientific investigations it will be observed that the Elberth-Gaffky bacillus is not identical with the bacillus calli. It will take further deffinite scientific proof to settle this question.

In all cases of doubtful diagnosis a bacteriological investigation by means of applying the "Widal Test" will determine the presence of absence of the baccillus typhosus. It has been the experience of bacteriologists that all culture media of the typhoid baccillus thrive best at body temperature of 98 2-5 or 37.0 C.

The Widal Serum Test for Typhoid Fever.

One part blood from a suspected case to 10 parts bouillion culture of typhoid baccilli. If culture is fresh and serum that of a person with typhoid fever, the baccilli collect in groups (called clumping), followed by mortillity of the bacteria.

The different types of typhoid are the ambutatary or walking typhoid. Hemorrhagic typhoid, para-typhoid and mixed infection of typhoid and malaria.

Typhoid fever is not contageous, but an infectious disease and always maintains a tendency to local limitations and is only altered by special conditions.

It is world wide in its distribution—no race, climate or altitude being exempt, and depends upon human activity for its distribution.

It has been observed in all civilized countries of the earth and the most varied climates and altitudes.

Typhoid fever costs the people of the United States alone, annually, $200,000,000. Reasoning from cause to effect, Georgia's pro rata part of this amount would be approximately $8,-782,222. This gives us an idea of how much money is expended for the suppression of typhoid fever to say nothing of the loss of thousand of valuable human lives and untold suffering it occasions.

This vast sum is the cost of negligence. With proper precaution the individual may almost surely escape infection, and by co-operation in city, town, country, state, and national government it could be stamped out by means of well organized state and national Public Health Boards.

Copper sulphate could be used on a large scale to destroy typhoid fever bacteria in large lakes, rivers, ponds and cesspools. Milk of lime could be used in the inner walls of houses, residents and tenements of all kinds.

First.—Apply general rules for the protection of entire cities or entire districts.

Second.—Apply special individual measures to stop the transmission of bacteria by carriers.

Third.—Preventive inoculation to make persons immune by the Wright and Netly vaccination method.

By this method persons are vaccinated against typhoid fever infection and rendered immune.

This method of rendering persons immune was first proved successful in the Boer War in South Africa, and is worthy of adoption by our state and National Health Boards. The period of incubation of the typhoid fever may vary from 10 to 20 days and may be accompanied by a chill, general malaise followed by fever.

Typhoid may vary from the mildest walking type to the severest malignant or hemorrhagic type or until death may close the scene.

The symptoms and pathological anatomy are very familiar to the profession. Complications of typhoid fever are as broad as the science of medicine itself. From the Peyers patches of the small intestines, the pathology may extend to the liver, brain and nervous system, lungs, spleen, kidneys, and joints.

Conservatism and fighting shy of all harmful drugs is the one great essential in the treatment of typhoid fever.

We are taking it for granted that the physician in charge has studied the action of all drugs he uses as his weapons of warfare. Also that he understands fully the family history temperaments and stamina of his patient.

An able and experienced physician and nurse is the key to the entire situation in a severe case of typhoid fever.

Poor nursing, underfeeding, venereal habits and unfavorable circumstances, render practice among colored people unsatisfactory. All cases of typhoid fever demanding treatment should be injoined to go to bed and remain quiet. Malignant and hemorrhagic cases should use a bed pan.

I very often give a teaspoonful every 4 hours of alcoholic or dycerinized solution (rendered neutral) of thymol, menthol, eucalyptol, salicylic, benzoic and boracic acid. In the interval between the above anteseptic mixture I have been very successful with 10 to 20 drops fl. ex. Echinacae, either with or without sulphate of strychnine.

Or varying the treatment I add 4 to 5 grains bismuth every 4 hours and antiseptic mixture between the bismuth.

If necessary echinacae and sulphate of strychnine every 6 hours.

The best haemostatic and "bug killer" is aromatic sulphuric acid,

Expistaxis intestinal hemorrhage and purpura hemorrhagica are controlled by sulphuric acid. Strychnine sulphate may be combined with it in the proportion 7 to 12 drops aromatic sulphuric acid and 1-16 gr. sulphate strychnine to the teaspoonful.

In blood poison, gangreen and typhoid fever, spirits turpentine stands at the head of all germicidal remedies. I have almost ceased using turpentine emulsion and depend on one thorough local application to the bowels, daily, in typhoid fever inflammation.

Eothol may be used for its antiseptic effect instead of echinacae.

In inflammations, peritonitis I use hot cloths, turpentine stupes, antiseptic plasters or cataplasms, vinegar and bran paultices. If the kidneys become ingorged I use the diuretics. For a depressed heart circulation attended with delirium, I use a combination of sulphate of strychnine, nitro-glycerine and digitalin. For intestinal hemorrhage I use large doses of bismuth internally. Morphine and atropine sulphate hypodermically to control the pain. Stimulants and nourishments to fortify against threatened collapse. If the cutaneous circulation is very weak, hot, dry surface with threatened enlarged glands of the throat, I sponge once or twice daily with a 45 or 50 per cent. solution of alcohol.

I never use subnitrate of bismuth and aromatic sulphuric acid at the same time, but alternate them. I withdraw the use of any drug which acts in any way unpleasant. In hemorrhagic conditions and inflammation of the bowels, I frequently resort to irrigation of the bowels, using normal salt solution or antiseptics enaemas. I do not use salts or Seidlitz powders, but prefer castor oil as a laxative. I do not use quinine sulphate as an antipyretic, but use it as a tonic in the convalescent stage. When I can watch its effects I sometimes use Zomakyne as a fever antispyretic. I keep a chart and have medicines given by the clock. I have my patients sponged or bathed from one to 3 times daily as conditions demand. It is useless to conclude that a barrel of butter milk will cure typhoid fever unless you add something to "kill bugs."

I use some one of the following diets and vary them according to the needs and appetites of my patients: Wild game, bird or chicken, or beef soups, sweet or butter milk, grape juice, blackberry juice, pepsin elixors, peptonoids, proteids meat-broths, eggs in combnation with alcohol, or gelatinous substances, flavored syrups, fruit juices of orange, lemon, raspberry, albumen.

In the year 1904, I attended 27 cases of typhoid fever near the village of Bowman, Ga. These cases varied from the mildest walking fever to the most malignant and complicated. About 5 cases developed nose bleed and intestinal hemorrhage. One case was mixed-traumatism and fever, which died, one case died from haematuria, one case died from septis and toxaemia with engorgement of the lung, one case died from malignancy. All hemorrhagic (intestinal) ipistoxis cases recovered. Out of a total of 27 cases only 4 died.

In the preparation of this article some reference was made by the writer to The Literary Digest, published by Funk & Wagnalls, New York, also Nathnogles Encyclopedia of Practical Medicine.

---

The Supreme Court of Georgia had just decided that when an employer summons a physician to care for an employe who has become suddenly ill, the employer is not liable for the bill unless there is a definite agreement between the employer and employe that the former shall be liable.

---

At the recent celebration of "Academic Day" of the University of Maryland it was decided to erect a tablet in memory of the late Major James Carroll, of the Army Medical Corps, who received his diploma from this university.

